# Tyrosine phosphorylation of myosin heavy chain during skeletal muscle differentiation: an integrated bioinformatics approach

**DOI:** 10.1186/1742-4682-2-12

**Published:** 2005-03-25

**Authors:** DF Harney, RK Butler, RJ Edwards

**Affiliations:** 1 Department of Clinical Pharmacology, Royal College of Surgeons in Ireland, 123 St. Stephens Green, Dublin 2, Ireland

## Abstract

**Background:**

Previously it has been shown that insulin-mediated tyrosine phosphorylation of myosin heavy chain is concomitant with enhanced association of C-terminal SRC kinase during skeletal muscle differentiation. We sought to identify putative site(s) for this phosphorylation event.

**Results:**

A combined bioinformatics approach of motif prediction and evolutionary and structural analyses identified tyrosines163 and 1856 of the skeletal muscle heavy chain as the leading candidate for the sites of insulin-mediated tyrosine phosphorylation.

**Conclusion:**

Our work is suggestive that tyrosine phosphorylation of myosin heavy chain, whether in skeletal muscle or in platelets, is a significant event that may initiate cytoskeletal reorganization of muscle cells and platelets. Our studies provide a good starting point for further functional analysis of MHC phosphor-signalling events within different cells.

## Introduction

Myosins, actin-based motor proteins, are expressed as multiple isoforms in all eukaryotic cells. They are oligomers consisting of one or two heavy chains to which one or more light chains are non-covalently attached. Myosins have been classified into 18 families based on the amino acid sequence differences in the N-terminal head domains, which contain highly conserved regions including actin- and nucleotide-binding sites [1,2]. The tail of myosin is the most variable domain and seems to be responsible for the specific role myosin plays in the cell.

Functional activities of most myosins such as actin-dependent ATPase activity or ability to move actin filaments *in vitro *are regulated in several ways, mainly by phosphorylation of the regulatory light chain, Ca^2+^-binding, or phosphorylation of the heavy chain [1,3] It has been previously claimed that the myosin heavy chain (MHC)undergoes tyrosine phosphorylation during insulin-mediated skeletal muscle differentiation, thus linking signal transduction to highly ordered myosin assembly [4]. Insulin modulates an association of myosin with C-terminal SRC kinase (Csk), a tyrosine kinase signalling molecule, and these interactions are fundamental in skeletal muscle differentiation. Although the claims of tyrosine phosphorylation of MHC *in vivo *remain somewhat controversial, tyrosine phosphorylation of non-muscle MHC IIa has also been implicated as an early event in human platelet activation [5]. To settle this controversy -and establish the role, if any, of MHC tyrosine phosphorylation it is important to identify sites at which such phosphorylation events may occur.

We have mapped potential phosphorylation sites on the skeletal muscle myosin heavy chain utilizing an integrated bioinformatics approach, supporting web-based motif predictions with evolutionary and structural data. Of all the sites analyzed in the bioinformatics approach, the data suggest Y163 and Y1856 as the leading candidates for insulin-mediated tyrosine phosphorylation.

## Methods

### Tyrosine Phosphorylation Predictions

Tyrosine phosphorylation site predictions were made with two different online resources using the sequences described below. NetPhos 2.0 produces neural network predictions based on sequence and structure [6]. Scansite predicts target motifs for different kinases using a positional selectivity matrix based on peptide library screening data [7,8] In addition, Scansite predictions were made for known phosphotyrosine recognition motifs for evidence of downstream signalling events. All Scansite predictions were made on the 'Low Stringency' setting to identify as many putative sites as possible. These sites were then supported or rejected on the basis of further analysis as described below.

### Evolutionary Analysis

Protein sequences for adult skeletal muscle myosin heavy chains (MYHSA) 1 and 2 were extracted from the SwissProt database [9] MYHSA1 [SwissProt : MYH1_HUMAN, P12882]; MYHSA2 [SwissProt ID: MYH2_HUMAN, Q9UKX2] and used as query sequences to extract closely related homologous proteins. First, BLAST [10] was used to search SwissProt-TrEMBL [9] and the known, novel and Genscan-predicted peptides of five EnsEMBL genome databases (Human, Mouse, Rat, Fugu, Zebrafish) [11] Redundant sequences were removed and ALIGN [12,13] was used to make pairwise alignments of each homologue with MYH1_HUMAN and to calculate the percentage identity across the entire length of the protein. Vertebrate homologues with at least 60% global identity were processed using an in-house homologue processing tool, HAQESAC [14]. Homologues were aligned using CLUSTALW [15] and badly-aligned sequences eliminated from the dataset. A neighbour-joining tree with 1000 bootstrap replicates was constructed using CLUSTALW and the sequences were grouped into subfamilies of orthologous proteins. The clade corresponding to skeletal muscle myosin heavy chains in Amniota (mammals, reptiles and birds) were then used as sequences for tyrosine phosphorylation motif prediction as described above.

### Secondary Structure Prediction

Secondary structure predictions were made for MYH1_HUMAN using the PSIPRED V2.3 website [16]. Because of the length of the protein, it was submitted in two overlapping chunks: residues 1–814 and 800 +.

### 3D Structure Analysis

3D structures were obtained from the Protein Data Bank (PDB) [17] and viewed with the RasMol viewer [18]. Three myosin heavy chain structures were identified: 2MYS, Chicken adult skeletal muscle myosin heavy chain; 1BR2, chicken gizzard smooth muscle myosin heavy chain; and 1B7T, *Aequipecten irradians *(Bay scallop) striated muscle myosin heavy chain. The corresponding SwissProt sequences [Swiss -Prot :2MYS: MYSS_CHICK, P13538]; [Swiss-Prot1BR2: MYHB_CHICK, P10587]; [Swiss -Prot1B7T: MYS_AEQIR, P24733] were downloaded and aligned with Human MYH1_HUMAN and MYH2_HUMAN using CLUSTALW. This alignment was used with the skeletal muscle myosin heavy chains (above) to assign putative tyrosine phosphorylation sites to their corresponding residues in the homologous 3D structures. Visualisation with RasMol and DSSP solvent accessibility data [19] was then used to infer whether potential sites of tyrosine phosphorylation were surface-exposed or buried.

## Results

**Figure 1:**
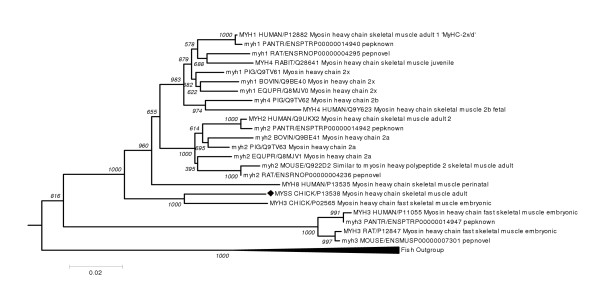
Neighbour-joining phylogeny of MHC homologues, with bootstrap support. PDB structure 2MYS is marked with a black diamond.

In total, twenty-three myosin heavy chain sequences were used for tyrosine phosphorylation motif prediction, which were divided into five groups of orthologous sequences (Figure 1). Important motifs are likely to be conserved during evolution and so we considered only those sites that were predicted to be phosphorylation motifs in all the sequences of at least one orthologous group. Because phosphorylation site predictors have a tendency to over-predict, we increased stringency by accepting only those motifs that received a NetPhos score of 0.8 or higher, or were predicted by both NetPhos and Scansite, in at least one human adult skeletal myosin heavy chain. This yielded fourteen putative sites (Table 1). Of these, six were predicted by both methods, including two motifs that were conserved across all sequences (MYH1_HUMAN Y163 and Y1856).

**Table 1:** Summary of predicted tyrosine phosphorylation sites.

Site^a^	NetPhos^b^							Scansite^c^							2D^d^	Surface Accessibility^e^			
	MYH1	MYH4	MYH2	MYH8	MYSS_CHICK	MYH3_CHICK	MYH3	MYH1	MYH4	MYH2	MYH8	MYSS_CHICK	MYH3_CHICK	MYH3	MYH1	1BR2	1B7T:A	2MYS: A	Mean
47	-	-	-	Y				-	-	-					E(4)	(14.5)	(21)	(16)	18.5
54	-	-	-	-	-	-	Y	-	-	-	-	-	-		E(7)		(52)	(60)	56.0
85	Y	Y	Y	Y	Y	Y	Y								C(2)	(34.7)	(28)	16	26.2
163	Y	Y	Y	Y	Y	Y	Y	YP	YP	YP	YP	YP	YP	YP	H(9)	13.3	47	8	22.8
286	Y	Y	Y	Y	Y	Y	Y			Y	Y				H(7)	(0)	0	0	0.0
313	Y	-						YP	-	Y	Y	YP	Y	Y	C(4)	7	5	8	6.7
413	Y	Y	Y	Y	-	Y	Y					-			E(7)			(68)	68.0
435						-	Y						-		H(9)	(0.7)	4	3	2.6
504	Y	Y	Y	Y	Y	Y	Y								H(9)	0	6	9	5.0
719	Y	Y	Y	Y	Y			YP	YP	YP	YP	YP	YP	Y	H(6)	(21.8)	19	9	16.6
1379	Y	Y	Y	Y	Y	Y	Y								H(6)				
1464	-	Y	-	Y	Y	Y	-	-		-				-	H(7)				
1492	Y	Y	Y	Y	Y	Y	Y				YP			P	H(6)				
1856	Y	Y	Y	Y	Y	Y	Y	Y	Y	Y	Y	Y	Y	Y	H(4)				

a. Sites are numbered relative to the MHC sequence MYH1_HUMAN/P12882.

b. Y indicates predicted tyrosine phosphorylation site in all the sequences of orthologous group, with a score of ≥ 0.8 in at least one human sequence. Dashes indicate lack of a tyrosine in that position.

c. Y indicates predicted tyrosine phosphorylation site in all the sequences of orthologous group on 'Low Stringency'. P indicates predicted phosphotyrosine recognition site in all the sequences of orthologous group on 'Low Stringency'. Dashes indicate lack of a tyrosine in that position.

d. PSIPRED (McGuffin, Bryson and Jones 2000) secondary structure position for MYH1_HUMAN. Letters indicate predicted secondary structure (H = helix, E = strand, C = coil). Numbers in brackets are confidence measures (0 = low, 9 = high).

e. Surface accessibility figures are "numbers of water molecules in contact with this residue *10, or residue water exposed surface in Angstrom**2" (Kabsch and Sander 1983). Missing values indicate residues missing from the PDB structure. Values in brackets indicate residues that are not tyrosines in the PDB structure.

To be phosphorylated, tyrosine residues must be accessible on the surface of the protein. Although the three-dimensional conformations of homologous myosin molecules will not be identical, the high degree of sequence conservation between human adult skeletal muscle myosin heavy chains and the three myosin sequences present in PDB allowed the inference of solvent accessibility. This was confirmed by the generally good agreement in surface accessibility measures both between models and between the different myosin chains of 1BR2 (data not shown). From these data, two sites (Y286 and Y435) were buried while a further two (Y313 and Y504) had very low solvent accessibility (Table 1). 3D data were not available for the four tyrosines in the C-terminal of the protein.

If tyrosine phosphorylation of MMHC-II is part of a signalling cascade, it is likely that some other protein will interact with the phosphotyrosine. We used Scansite to look for phosphotyrosine interaction motifs and found three SH2 domain recognition motifs that matched potentially exposed phosphorylation sites. Because Scansite also identifies the interacting protein, we interrogated the Gene Cards [20] entry for each kinase and SH2 domain for expression patterns. Only four kinases and two SH2 domains had evidence from UniGene [21] or SAGE [22] of expression in skeletal muscle, while only one kinase (INSR) and one SH2 domain (PIK3R1) had evidence from both (Table 2). Interestingly, both of the latter pair were predicted to interact with the same, totally conserved, motif (MYH1_HUMAN Y163). Furthermore, both are involved in insulin-mediated pathways (see Discussion). Two kinases expressed in skeletal muscle were predicted to interact with Y1856. These were an SRC kinase and ABL1, which interacts with SORBS1 following insulin stimulation [23].

**Table 2:** Interacting enzymes predicted by Scansite.

Site^a^	Enzyme^b^	Gene Card	UniGene^c^	SAGE^c^	Full Name
163	EGFR Kinase	EGFR	Yes	No	EGFR (epidermal growth factor receptor (erythroblastic leukemia viral (v-erb-b) oncogene homolog, avian))
	Insulin Receptor Kinase	INSR	Yes	Yes	INSR (insulin receptor)
	p85 SH2	PIK3R1	Yes	Yes	PIK3R1 (phosphoinositide-3-kinase, regulatory subunit, polypeptide 1 (p85 alpha))
	Shc SH2	SHC1	No	No	SHC1 (SHC (Src homology 2 domain containing) transforming protein 1)

286	EGFR Kinase	EGFR	Yes	No	EGFR (epidermal growth factor receptor (erythroblastic leukemia viral (v-erb-b) oncogene homolog, avian))

313	EGFR Kinase	EGFR	Yes	No	EGFR (epidermal growth factor receptor (erythroblastic leukemia viral (v-erb-b) oncogene homolog, avian))
	Fgr Kinase	FGR	No	No	FGR (Gardner-Rasheed feline sarcoma viral (v-fgr) oncogene homolog)
	PDGFR Kin	PDGFRB	No	Yes	PDGFRB (platelet-derived growth factor receptor, beta polypeptide)
	Itk SH2	ITK	No	No	ITK (IL2-inducible T-cell kinase)
	Fgr SH2	FGR	No	No	FGR (Gardner-Rasheed feline sarcoma viral (v-fgr) oncogene homolog)

719	Lck Kinase	LCK	No	No	LCK (lymphocyte-specific protein tyrosine kinase)
	Abl Kinase	ABL1	No	Yes	ABL1 (v-abl Abelson murine leukemia viral oncogene homolog 1)
	Itk SH2	ITK	No	No	ITK (IL2-inducible T-cell kinase)
	Src Kinase	SRC	Yes	No	SRC (v-src sarcoma (Schmidt-Ruppin A-2) viral oncogene homolog (avian))

1492	Src Kinase	SRC	Yes	No	SRC (v-src sarcoma (Schmidt-Ruppin A-2) viral oncogene homolog (avian))
	Lck SH2	LCK	No	No	LCK (lymphocyte-specific protein tyrosine kinase)
	Fgr SH2	FGR	No	No	FGR (Gardner-Rasheed feline sarcoma viral (v-fgr) oncogene homolog)
	Src SH2	SRC	Yes	No	SRC (v-src sarcoma (Schmidt-Ruppin A-2) viral oncogene homolog (avian))

1856	Lck Kinase	LCK	No	No	LCK (lymphocyte-specific protein tyrosine kinase)
	Src Kinase	SRC	Yes	No	SRC (v-src sarcoma (Schmidt-Ruppin A-2) viral oncogene homolog (avian))
	Abl Kinase	ABL1	No	Yes	ABL1 (v-abl Abelson murine leukemia viral oncogene homolog 1)

a. Sites are numbered relative to the MHC sequence MYH1_HUMAN/P12882.

b. Enzyme identified by Scansite.

c Skeletal muscle expression data from GeneCards (Rebhan et al. 1997).

## Discussion

Bioinformatics alone cannot identify a functional motif; supporting experiments will always be needed for conclusive evidence. Nevertheless, while other sites cannot be categorically excluded, the combined data presented here identify Y163 and Y1856 as the most likely sites for tyrosine phosphorylation events in skeletal muscle. Both NetPhos and Scansite predicted these motifs for all mammalian adult skeletal myosin heavy chain sequences analysed, indicating strong evolutionary conservation (Figure 1). A kinase predicted to be responsible for phosphorylation of each site is expressed in skeletal muscle, as was an SH2 domain protein that was predicted to interact with a phosphotyrosine at Y163. Analysis of predicted secondary structures and homologous 3D structures indicates that these sites may be accessible on the protein surface. The position of Y163 as part of an alpha helix within the globular myosin head domain does raise concerns that it is potentially difficult to phosphorylate, even though it is on the surface of the domain. Nevertheless, depending on the relative conformations of the solved *in vitro *chicken myosin structures compared to *in vivo *human myosin, Y163 might still be available for phosphorylation. Y1856 is in region of low predicted secondary structure (Table 1), indicative of a flexible loop region more usually associated with phosphorylation sites.

Myosin heavy chain (MHC) undergoes tyrosine phosphorylation during insulin-mediated differentiation in skeletal muscles and the degree of phosphorylation increases in line with differentiation [4]. Interestingly, for the strongest candidate tyrosine phosphorylation site, Y163, both the kinase and interacting SH2 domain predicted by Scansite are involved in insulin-mediated pathways. The kinase INSR is a transmembrane receptor that binds insulin [24] while the SH2 domain protein PIK3R1 is necessary for the insulin-stimulated increase in glucose uptake and glycogen synthesis in insulin-sensitive tissues [25]. We can therefore conclude that Y163 remains a strong candidate site for insulin-mediated tyrosine phosphorylation of myosin heavy chain, despite concerns over accessibility.

As phosphorylation sites are often in the tails of proteins, the tyrosines outside the main globular domains, namely Y1379, Y1492 and Y1856, are also potential candidates for phosphorylation. The strongest of these is Y1856, which is both C-terminal and predicted to be phosphorylated by the kinases SRC and ABL1, which are found in skeletal muscle (Table 2). Perhaps of most interest is ABL1, a protein known to be associated with "Sorbin and SH3 domain containing 1" (SORBS1) during insulin signalling in other cell lines [23]. SORBS1 is highly expressed in skeletal muscle (data not shown) and is involved in formation of actin stress fibres and focal adhesions; its orthologue, CAP, has been identified as an important adaptor during insulin signalling in mice [26-28]. Furthermore, the SORBS1 gene has been implicated in the pathogenesis of human disorders with insulin resistance [29].

Csk has been shown to be associated with the hormone 1, 25(OH)_2_-vitamin D_3 _resulting in the stimulation of the growth-related mitogen-activated protein kinase (MAPK). The phosphorylated form of MAPK is then translocated to the nucleus where it induces the expression of c-myc oncoprotein associated with skeletal muscle proliferation [30]. In addition, Csk has also been implicated in the regulation of integrins and the control of cell attachment and shape [31] Goel et al. showed that insulin can phosphorylate myosin, leading to an association with Csk and thus to a decrease in c-Src activity. This has also been shown in fibroblast cell lines following stimulation of the insulin-like growth factor-I receptor [32]. This demonstrates the potential for skeletal muscle differentiation after phosphorylation of Y163 and/or Y1856 of the MHC.

Harney et al. have shown that non-muscle myosin heavy chain type IIA in platelets undergoes tyrosine phosphorylation and subsequent dephosphorylation in a time-dependent manner [5]. In common with other cells, the cytoskeleton of platelets comprises actin filaments, microtubules and myosin molecules. Myosins form rings within the platelet that maintain a spherical shape and several lines of evidence suggest that these rings reorient following platelet activation to permit spreading [33,34]. While myosin function therefore appears critical to platelet spreading, studies using cytoskeleton inhibitors have shown that at least the early events of platelet activation are not dependent on the cytoskeletal changes [35]. Our work is suggestive that tyrosine phosphorylation of myosin heavy chain, whether in skeletal muscle or in platelets, is a significant event that may initiate cytoskeletal reorganization of muscle cells and platelets. Our studies provide a good starting point for further functional analysis of MHC phosphor-signalling events within different cells.

### Supplementary Information

Full prediction results, sequence alignments and links to in-house software used can be found at: 

## Competing interests

The author(s) declare that they have no competing interests.
